# Longitudinal extensive transverse myelitis (LETM) in children: A twenty-year study from Oman

**DOI:** 10.17712/nsj.2017.2.20160352

**Published:** 2017-04

**Authors:** Roshan Koul, Amna M. Alfutaisi, Renjith Mani, Rana A. Abdel Rahim, Dilip K. Sankhla, Faisal M. Al Azri

**Affiliations:** *From the Department of Child Health and Radio Diagnosis, College of Medicine and Health Sciences, Sultan Qaboos University, Muscat, Oman*

## Abstract

**Objective:**

The data on children with diagnosis of idiopathic transverse myelitis (ITM) was searched to find the pattern of myelitis in Oman.

**Method:**

A retrospective study was carried out from January1995 to December 2014. Electronic medical records and patient medical files were seen to get the complete data of the children with ITM. This work was carried out at Sultan Qaboos University hospital, Muscat, Oman. The ethical committee of the hospital had approved the study. The diagnosis was based on the established criteria. Other causes of myelopathy were excluded.

**Results:**

19 children with idiopathic transverse myelitis were found. There were 18 out of 19 (94.6%) children with longitudinal extensive transverse myelitis (LETM).

**Conclusion:**

Longitudinal transverse extensive myelitis is the most common form of ITM in Oman.

Acute transverse myelitis (ATM) presents with acute onset paraplegia or quadriplegia with sensory and autonomic involvement.^1^ The weakness progresses in initial few days with maximal weakness reached in 3 to 4 days most of the times. The illness commonly presents following an upper respiratory infection. Acute myelitis may be a part of acute disseminated encephalitis (ADEM) or the first presentation of primary demyelination like multiple sclerosis and neuromyelitis optica.^1^ The ATM has to be differentiated from compressive and other non-inflammatory causes of myelopathy for treatment and prognosis point of view.^1^ Certain viral infections, paraneoplastic syndromes and specific autoimmune diseases may involve spinal cord. Subtypes of ATM are acute partial or complete and transverse/segmental or longitudinal depending upon the severity and number of vertebral segments involvement respectively. Longitudinal extensive transverse myelitis (LETM) is defined as more than 3 consecutive vertebral segments involvement on MRI.^2^ ATM is a disease more often seen in adults and approximately 20 percent cases are under age of 18 years^1^,^3^ Preceding illnesses and vaccination have been reported in 47% and 28% cases respectively.^4^ This study was carried out to find out the pattern of ITM in this country.

## Methods

Sultan Qaboos University hospital is a tertiary care center of the country. This hospital also is the final referral center for all cases of acute flaccid paralysis (AFP). The cases seen at this hospital represent the entire country cases. Acute transverse myelitis formed a part of acute flaccid paralysis (AFP) program to eradicate poliomyelitis in Oman. All the children below 15 years age with AFP in the country were referred to this hospital. A retrospective study was carried out from January 1995 to December 2014. The hospital Ethical Committee approved this study. The diagnosis of idiopathic transverse myelitis (ITM) was based on established criteria.^5^ All the inpatient and electronic medical records were reviewed. The children with final diagnosis of ITM at the time of the discharge were included in the study. The diagnosis was based on established criteria and exclusion of the other causes on detailed neurophysiological tests, cerebrospinal fluid (CSF) examination, MRI brain and spine. The CSF was sent for routine cytology, biochemistry and viral screening (entero viruses, mumps, varicella zoster virus, cytomegalovirus, and herpes). The CSF analysis for aquaporin antibodies was sent in patient with compatible diagnosis of neuromyelitis optica after 2006. Baseline blood work up like ESR, peripheral blood film, antinuclear antibody were carried out in all. Methylprednisolone (30 mg/kg/day for 5 days followed by oral prednisolone for 4 weeks and then taper over next 2 weeks was used in all. Additional intravenous immunoglobulin was used in 12 children. Two children underwent plasmapheresis when methylprednisolone did not improve the weakness. All the children were followed up in the Pediatric Neurology Outpatient Clinic.

## Results

**Figure 1:**
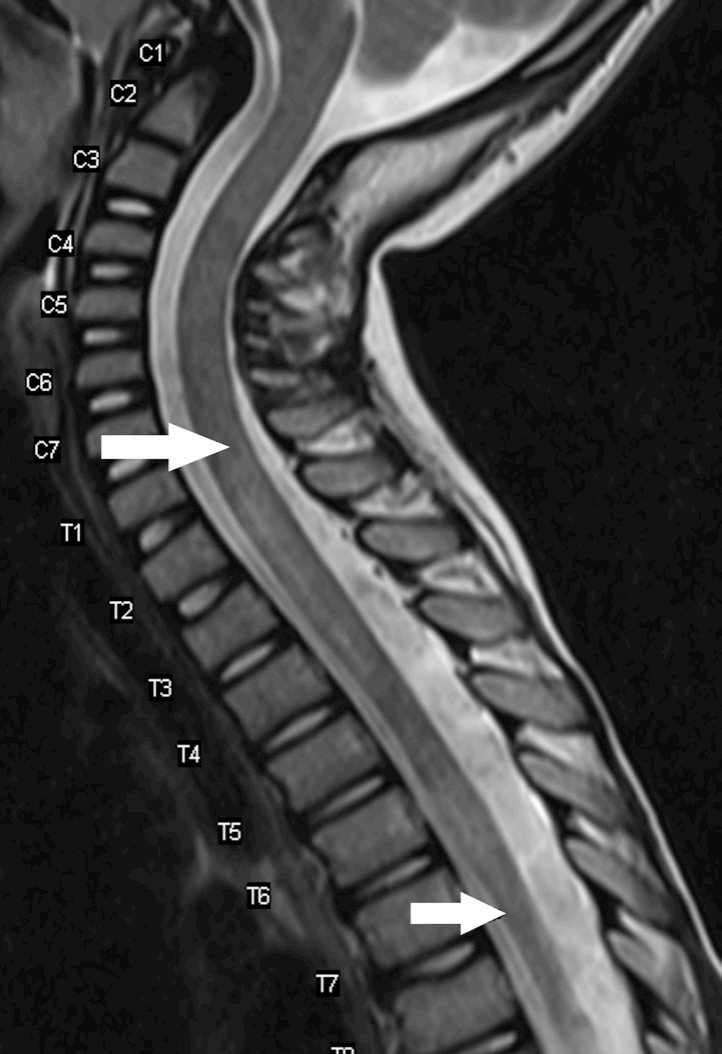
- T2 weighted sagittal MRI spine shows long segment transverse myelitis from C7 to T7 level. (arrows)

**Figure 2:**
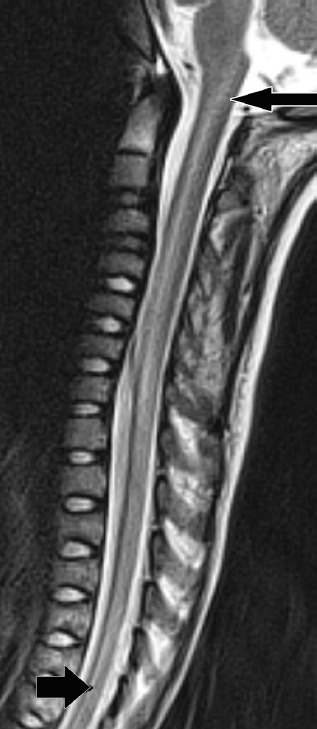
- T2 weighted sagittal MRI spine shows long segment transverse myelitis from C1 to T7 level. (arrows)

Twenty-four children presented with features of acute TM and 5 children were excluded from the study. These were 3 cases of neuromyelitis optica, one each case of spinal arteriovenous malformation and vasculitis. The children with final diagnosis of ADEM were not included in the study. Nineteen (11F: 8M) children had idiopathic transverse myelitis ITM. The youngest patient was a 5 month old child. All the children were in the age group of 5 months to 11.8 years. Mean age was 6.4 years and 7 children were below 5 years of age. The site of lesion was cervical spinal cord in 2 (10.5%), thoracic in 4 (21%) and cervicothoracic in 13 (68.5%). One child had entire length of the spinal cord involvement. All but one had more than 3 vertebral segments involvement fitting the diagnosis of longitudinal extensive TM. (**Figure 1 & 2**). The CSF was examined in 11 children. The cell count ranged between 0 and 165 with a mean of 20 cells/cumm, 5 children had no cells, and 4 children had less than 10 cells. The CSF proteins were mildly raised in 2 children only. Oligo clonal bands and viral studies were negative in all. Sixteen children were on regular follow up and 3 were lost to follow up. A complete recovery was seen in 5 children (31.2%), 3 children (18.7%) were left with mild deficit while 8 children had severe neurodeficit (68.7%).

## Discussion

The diagnosis of ITM was based on clinical features, myelogram and CT myelography in the past. Contrast blocks, spinal cord swelling (expansion) were the features to find the level and extent of myelitis. Transverse label was to denote the sensory level felt across the trunk.^1^ With advent of MRI exact level and extent of involvement of spinal cord was possible. A clear proportion of segmental/transverse myelitis and LETM is not available in the ITM cases. Our data suggests LETM to be the most common form of ITM in this region. Eighteen cases out of 19 (94.7%) had LETM. Several reports observed that the LETM was typically seen in children with ADEM.^1^,^6^,^7^ However none of our LETM had features of ADEM, though such an association of LETM was seen in children with ADEM, a separate group reported earlier.^8^ All the children did not undergo CSF examination. The study was allowed in 11 cases only. In this country performing a lumber puncture still has social restrictions. CSF features were typical of idiopathic transverse myelitis in all.

Spinal cord level and extent of involvement also varies in different reported series.^7^ In a case series of 47 children with ITM, 50% had cervical lesion, 40% had thoracic level lesion.^3^ In another series of 170 patients, MRI abnormality was in cervical region in 44% and thoracic in 37%.^4^ However in our patients pattern of MRI involvement was different to these reports. It was cervical in 2 (10.6%), thoracic in 4 (21.2%) and cervicothoracic in 13 (68.2%). The pattern in our series was predominant long segment TM, different from the previously reported series.^3^,^4^,^7^ The underlying mechanism for such a pattern was difficult to explain on geographical conditions alone. A possible reason could be longitudinal extensive TM involving a large length of the spinal cord due to genetic predisposition in this country.

The ADEM and neuromyelitis optica have to be excluded before a diagnosis of ITM is made. There is involvement of spinal cord in children with ADEM. This was observed in a separate study on ADEM in children and previously reported.^8^ Neuromyelitis optica (NMO) is an autoimmune condition affecting optic nerves and spinal cord. Aquaporin-4 antibody damages the optic nerves and spinal cord.^9^ NMO was excluded in children who presented after 2006. It is possible that children before this might have been missed as aquaporin antibody assay was not available. Three children with confirmed diagnosis of NMO were excluded from this study.

Methylprednisolone is the mainstay in the treatment in ITM.^10^ Methylprednisolone was used in all, IVIG was given in 12 and 2 cases underwent plasmapheresis. Ventilatory support was required in 3 (16%) cases as these children had severe respiratory muscle weakness. An old principle of one third complete recovery, one third partial recovery and one third very poor recovery usually holds true in many series of ITM but not in all.^1^,^10^,^11^ A rapid progression of symptoms, high level of deficit, spinal shock are the bad prognostic factors.^11^,^12^ Complete follow up was available in 16 cases and 3 cases were lost to follow up. Five (31.3%) children recovered completely, 3 (18.7%) had minor deficit and 8 (50%) had poor outcome. The outcome in our series was significantly poorer as compared to the studies indicating 10-20% poor outcome.^12^,^13^ The ITM was a serious spinal cord disease in children and the incidence was approximately one case per million children population per year in Oman. This incidence was an indirect one derived from the fact that 19 cases were seen over twenty years in a country with a population of approximately one million under 15 years age. Our limitation of the study was NMO cases which could have been missed prior to 2006 as aquaporin antibody assay was not available.

In conclusions, longitudinal extensive transverse myelitis (LETM) is the most common form of idiopathic transverse myelitis in children in Oman. It is worthwhile to study ITM in children in other Gulf countries.
